# Effect of CAD/CAM Guide Plate Combined With Socket-Shield Technique in Immediate Implantation of Anterior Teeth Aesthetic Area and Its Influence on Aesthetics

**DOI:** 10.3389/fsurg.2021.833288

**Published:** 2022-01-25

**Authors:** Zhigang Wang, Jianwei Liu, Xinquan Wang, Ning Wang, Min Teng

**Affiliations:** ^1^Department of Stomatology, Zibo Central Hospital, Zibo, China; ^2^Department of Stomatology, People's Hospital of Changle County, Weifang, China; ^3^Department of Stomatology, Rizhao Traditional Chinese Medicine Hospital, Rizhao, China; ^4^Department of Stomatology, Tengzhou Central People's Hospital, Zaozhuang, China

**Keywords:** immediate implantation, anterior teeth, CAD/CAM guide plate, socket-shield technique, aesthetics

## Abstract

**Purpose:**

The purpose of this study is to discuss the effect of computer-aided design or computer-aided manufacturing (CAD/CAM) guide plate combined with socket-shield technique (SST) in immediate implantation of anterior teeth aesthetic area and its influence on aesthetics.

**Methods:**

A total of 102 patients with immediate implantation in our hospital from March 2017 to March 2020 were selected. According to different repair methods, patients were divided into conventional group (*n* = 51) and observation group (*n* = 51). Traditional immediate implantation was performed in conventional group. The observation group underwent immediate implantation with CAD/CAM guides combined with SST. Immediately after operation and 12 months after operation, the success rate, implant deviation, periodontal index, absorption of labial bone plate, complications, aesthetic effects, and satisfaction of the two groups were observed.

**Results:**

There was no significant difference in the success rate between the two groups (*p* > 0.05). The implant deviation values in the observation group were all lower than those in the conventional group (*p* < 0.05). PD, PLI, and SBI in the observation group were all lower than those in the conventional group (*p* < 0.05). The absorption value of labial bone plate in the observation group were all lower than those in the conventional group (*p* < 0.05). The total incidence of complications in the observation group (5.88%) was lower than that in the conventional group (19.61%) (*p* < 0.05). The PES and WES in the observation group were higher than those in the conventional group (*p* < 0.05). The total satisfaction in the observation group (92.16%) was higher than that in the conventional group (76.47%) (*p* < 0.05).

**Conclusion:**

**:** The application of CAD/CAM guide plate combined with SST in immediate implantation of anterior teeth aesthetic area has a good effect, which can improve the accuracy of implantation, improve the periodontal environment, reduce bone resorption, reduce complications, improve aesthetics, and have high patient's satisfaction.

## Introduction

The aesthetic area of anterior teeth refers to any tooth-alveolar part that can be seen when laughing. Because of tooth defects, pulp diseases, trauma, and other factors, this area is easily damaged, which will greatly affect the patient's appearance, occlusion, pronunciation, physiological stimulation, and other functions and then affect the patient's mental health and social life (1). Immediate implantation is an oral repair technique that implants are implanted into the bone immediately after tooth extraction. It has the advantages of less trauma, short period, and fewer operations and can reduce the pain of patients and the social and economic burden of patients. It has been widely accepted by patients and doctors (2). The aesthetic area of anterior teeth, as an important position in oral cavity, has a special anatomical structure, and patients often have higher aesthetic requirements for this area. Therefore, it is undoubtedly a great challenge for most doctors to immediate implantation of aesthetic area of anterior teeth. The three-dimensional position and axial direction of the implant are the prerequisites for obtaining the long-term stability of the implant. However, the traditional implant operation mainly relies on the personal experience of doctors, which easily leads to the deviation between the implant position and the preoperative design position.

With the continuous development of information technology, computer-aided design or computer-aided manufacturing (CAD/CAM) is widely used in daily life. In 1970s, the team of French scholar Duret first introduced CAD/CAM technology into the design and manufacture of dental prostheses (3). The CAD/CAM technology uses CBCT data to reconstruct jaw information through professional software, designs and makes implant guide plate using CAD/CAM, and then guides each reaming drill and implant placement during implant operation (4). CAD/CAM guide plate can accurately control the three-dimensional direction of the implant, reduce the operation risk, shorten the operation time, and greatly promote the development of digital dental implant technology.

Clinical studies and animal experiments show that immediate implantation after tooth extraction cannot avoid vertical and horizontal bone resorption of surrounding bone tissue, especially the absorption of labial side, and the alveolar bone of labial side will collapse rapidly, resulting in alveolar ridge stenosis, which will adversely affect the healing of soft tissue, and then, it is difficult to achieve the ideal aesthetic requirements (5). In recent years, how to preserve alveolar bone with better repair methods has become a hot issue in the clinic. In the 1960's, root penetration technology became popular, mainly used to prevent alveolar bone resorption (6). In 2010, Hürzeler created the socket-shield technique (SST) based on the root penetration technique. The key points of this method are incomplete extraction of the affected teeth, preservation of cementum and periodontal ligament of labial dental tablets, and implantation of implants in the palatal side, which can reduce the risk of absorption of labial bone plate, resulting in long-term preservation of the alveolar ridge contour (7). Dayakar's team reported that the blood supply of periodontal membrane between tooth piece and buccal bone tissue was good, but no obvious absorption and reconstruction of labial bone plate were found. After the application of SST, there was new cementum between the implant and the retained root slice, which formed osseointegration. This technique is a good plan for implant restoration in the aesthetic area (8).

The implementation of SST has high technical sensitivity. As influenced by the improper operation of the operator and complicated anatomical structure, it is easy to cause the loosening and displacement of the labial dental shield, and even break and fall off, which leads to the failure of retaining the dental shield. In addition, there is a high probability of complications such as labial gingival recession after immediate implantation, and only patients with a thickness of the labial bone plate >1 mm can achieve better aesthetic results. Therefore, this research carried out SST based on the CAD/CAM guide plate, to provide some reference for clinical oral restoration.

## Materials and Methods

### Research Object

A total of 102 patients with immediate implantation from March 2017 to March 2020 were selected. Inclusion criteria were as follows: age ≥18 years; single anterior tooth has no retention value; complete labial bone plate, thickness of labial bone plate ≥1 mm; the gingival biotype was a thick gingival type; periodontal tissue was normal, and there was no obvious inflammation at the root tip; do not smoke; good oral hygiene; no mouth opening limitation, the distance between the incisal edges of the upper and lower central incisors was <3.7 cm; can tolerate operation; informed consent to the study. Exclusion criteria were as follows: systemic diseases with contraindications for operation (such as leukemia, hemophilia, osteoporosis, systolic blood pressure >180 mmHg or diastolic blood pressure >100 mmHg in patients with hypertension, fasting blood glucose >8.88 mmol/L in patients with diabetes); accompanied by night bruxism, clenching teeth and other bad oral habits; mental disorders, unable to cooperate with the investigator; pregnancy or lactation; lost to follow-up or dropped out halfway. According to different repair methods, patients were divided into conventional group (*n* = 51) and observation group (*n* = 51). General data were equally comparable between the two groups (*p* > 0.05). See Table 1 for specific general information.

**Table 1:** Comparison of general data between the two groups (n, %, $\bar{x}\pm$s).

**Group**	**Gender**		**Average age (years)**	**Missing tooth position**		**Causes of dental disease**		
	**Male**	**Female**		**Loss of upper anterior teeth**	**Loss of lower anterior teeth**	**Lesions of tooth and Pulp**	**Tooth trauma**	**Other**
Conventional group (*n* = 51)	25 (49.02%)	26 (50.98%)	40.67 ± 8.52	34 (66.67%)	17 (33.33%)	28 (54.90%)	17 (33.33%)	6 (11.76%)
Observation group (*n* = 51)	28 (54.90%)	23 (45.10%)	41.39 ± 8.06	33 (64.71%)	18 (35.29%)	30 (58.82%)	14 (27.45%)	7 (13.73%)
*χ^2^/t-*value	0.353		0.438	0.043		0.436		
*p*-value	0.552		0.662	0.835		0.804		

### Research Methods

Preoperative preparation: preoperative oral examination, imaging examination, and routine biochemical examination were carried out, and oral health education was carried out. Oral examination includes the followings: local soft and hard tissues of missing teeth, missing tooth space, occlusal relationship, periodontal condition of adjacent teeth, periodontal health of the whole mouth, joint condition, etc. It determined the implant plan and introduces the implant type, operation process, and possible complications to patients in detail. Imaging examination includes the followings: CBCT imaging, assessment of available bone width and bone height at the missing tooth, and adjacent to important anatomical structures.

Intraoperative operation: ① Traditional immediate implantation was performed in conventional group: As conventional disinfection and towel spreading, after local infiltration anesthesia, the implant cavity was prepared on the palatal side of the alveolar fossa. After the preparation of the hole was completed, a large amount of normal saline were used to flush the implant cavity and implanted the implant (Nobel Biocare, Sweden). The cover screws or healing abutments were placed, Bio-Oss bone powder (Geistlich, Switzerland) was filled into the labial jump gap of the implant, Bio-Gide barrier membrane was covered, and intermittent sutures were performed. ② The observation group underwent immediate implantation with CAD/CAM guides combined with SST: the data derived from CBCT were imported into Simplant Pro 2011 software (Materialize, Belgium), the target area was selected, the images of upper and lower dentition and bone tissue were extracted, and the high-quality three-dimensional jaw model was reconstructed. According to the condition of jaw and the position of prosthesis, the available bone width and available bone height in the missing tooth area were measured on the sagittal image, so as to determine the three-dimensional position, type, and size of the implant and design the surgical guide plate with accuracy exceeding millimeter level for patients. Finally, the CAD/CAM dental implant guide plate was manufactured using the dental guide plate forming machine. The upper and lower dentition models of patients were made with silicone rubber, and the super anhydrite model was poured and sent to the processing center. Conventional disinfection and towel spreading, after local infiltration anesthesia, SST was started. First, the labial apical fenestration, root removal, and alveolar fossa were performed. A U-shaped incision was made on the side of the lip from the incisor of the natural tooth, and the full-thickness flap was turned to expose the alveolar bone of the apical tooth. The labial side window was opened by high-speed turbine, and the root of the apical tooth was removed. The socket was cleaned with a spoon, and the socket was rinsed with plenty of saline. High-speed turbines were used to divide the root in the near and far directions to avoid alveolar bone damage. After the labial-palatal root was completely separated, the palatal root was removed, the labial-palatal slice was retained, the thickness was adjusted to 1 mm, and the shield shape was formed to 2 mm subgingival. The CAD/CAM guide plate prepared preoperatively was fixed in the dentistry, and the implant socket was prepared following the guide hole of the guide plate and implanted the implant (Nobel Biocare, Sweden). There was a 2-mm distance between the implant and the tooth piece. Bio-Oss bone powder (Geistlich, Switzerland) was filled into the labial jump gap of the implant, Bio-Gide barrier membrane was covered, and intermittent sutures were performed.

Postoperative treatment: after implantation, two groups of antibiotics were given for 3–5 days, 3 times a day, and two groups of chlorhexidine gargle were given for 14 days, 3 times a day. After 6 months of implantation, the superstructure was repaired, CBCT was taken to confirm the osseointegration of the implant, and the final model was taken for a permanent repair. All immediate implant operations were performed by the same operator. Patients were ordered to have regular checkups and informed of precautions for oral health maintenance. Patients in both groups were followed up for 12 months and the outcomes were evaluated. All evaluation items were completed by the same physician who did not participate in the operation.

### Observation Index

After 12 months of operation, the success rate of implant was evaluated according to Albrektsson standard (9), and the successful evaluation criteria were as follows: ① Implants did not loosen or fall off; ② No persistent or irreversible symptoms of the implant, including paresthesia, infection, pain, inflammation, etc.; ③ Imaging showed no transmission zone around the implant; ④ Implant neck bone resorption <2 mm in the first year and <0.2 mm every year thereafter.

Immediately after operation, the oral cavity of the two groups was scanned by the intraoral scanner to obtain the data information of the oral implants, and the image information was imported into the computer software for registration. The shoulder deviation, root deviation, depth deviation, and angle deviation between the preoperation design and the postoperation implant were measured for 3 times and averaged.

After 12 months of operation, the periodontal index was detected by periodontal probe, including 6 sites of each tooth: the mesial, center, and distal of the buccal, and the mesial, center, and distal of the tongue. ① Probing depth (PD): the distance from the gingival margin to the bottom of the pocket or the bottom of the gingival groove was measured with a periodontal probe, and PD of healthy gingival was <2–3 mm. ② Plaque index (PLI): patients were instructed to gargle plaque for 2 min, blow-dry the tooth surface, and then lightly scratch the tooth surface with a probe. According to the amount and thickness of plaque on the tooth surface, the score method of 0–3 points was used. The higher the score, the more plaque. ③ Sulcular bleeding index (SBI): the periodontal probe was gently inserted into the gingival sulci, the probe was removed 30 s later, the gingival bleeding was observed, and the score method of 0–5 points was used. The higher the score, the greater the bleeding tendency.

Immediately after operation and 12 months after operation, the same doctor used the same CBCT to take photos of the two groups under the same parameters. The parameters were set as follows: the shooting voltage was 120 kV, the current was 5 mA, the effective exposure time was 8.9 s, and the resolution was 0.4 mm. Every time the patient took pictures, patients were sat upright, the occlusal plane was parallel to the horizontal plane, and the middle line was perpendicular to the horizontal plane. The built-in software KaVo-eXam Vision was used to measure the multilevel labial bone plate thickness of 1, 3, and 5 mm of the root of the implant shoulder (IS). They were denoted as 1, 3, and 5 m-IS, respectively, the measurement images passed through the center of the implant, and the measurement accuracy was 0.01 mm and measured for 3 times and averaged. Labial plate absorption value = immediate postoperative labial plate thickness – 12 months postoperative labial plate thickness.

The complications that occurred in the two groups within 12 months after operation were recorded, including periimplant inflammation, crown fracture, gingival swelling, neuropathic pain, malocclusion, and other complications.

After 12 months of operation, the photos of the aesthetic area of the anterior teeth were taken, and the aesthetic effect was evaluated according to the pink aesthetic index (PES) and the white aesthetic index (WES). The score was obtained by comparing with the tooth of the same name on the opposite side. There were 12 members in the scoring group, who came from the Department of Stomatology, Implant Department, and Imaging Department, and the final score was scored by the total average. ① PES includes gingival margin level, alveolar process shape, soft tissue texture, soft tissue shape, soft tissue color, distal gingival papilla, and proximal gingival papilla, with a score of 0–2 points, with a full score of 14 points. The higher the score, the better the aesthetic effect of soft tissue. ② WES includes crown shape, crown outline, crown color, surface texture, and transparency, with a score of 0–2 points, with a full score of 10 points. The higher the score, the better the aesthetic effect of the restoration.

After 12 months of operation, the self-made questionnaire was used to evaluate the satisfaction of the two groups, including the overall appearance, occlusal ability, stability, comfort, etc. The total score was 100 points; 80–100 points denote very satisfied, 60–79 points denote satisfied, and <60 points denote dissatisfied. Total satisfaction = (very satisfied+satisfied)/total number of cases ×100%. The content validity index of our self-made questionnaire was 0.85, and the internal consensus reliability Cronbach's α coefficient was 0.779.

### Statistical Methods

SPSS 22.0 software was used for the analysis, measurement data were expressed as mean ± standard deviation, and *t*-test was used to analyze the comparison. Count data were expressed as a ratio, and chi-square test was used to analyze the comparison. *p* < 0.05 was statistically significant.

## Results

### Comparison of Clinical Efficacy Between Two Groups

**Figure 1:**
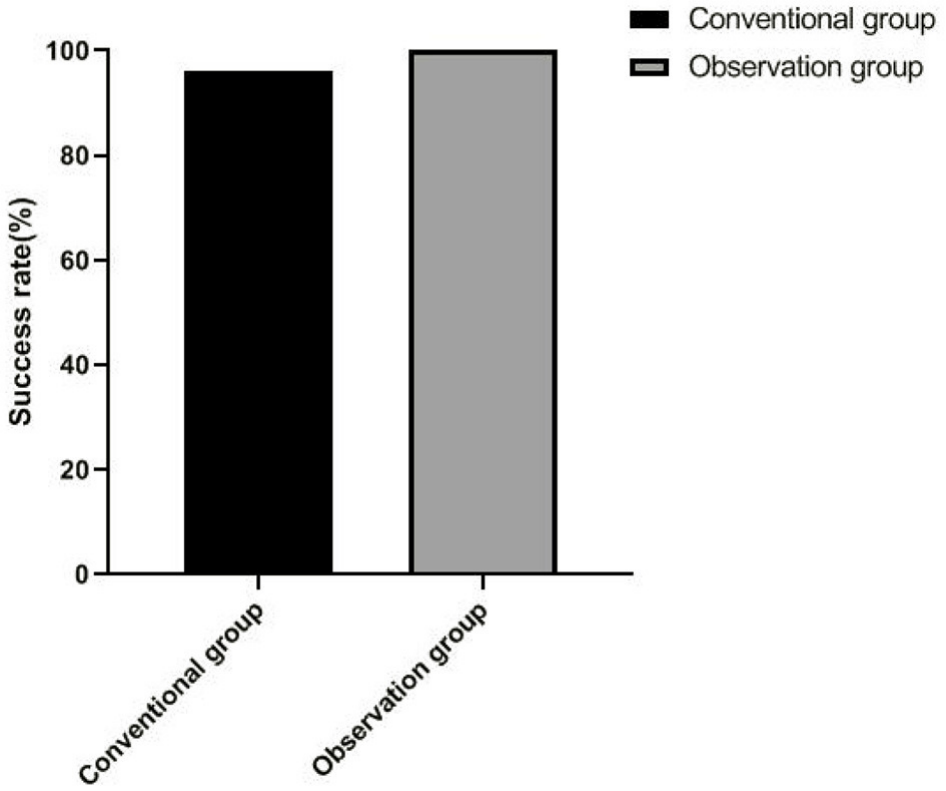
Comparison of clinical efficacy between two groups.

There were 49 successful cases and the success rate was 96.08% in the conventional group, and there were 51 successful cases and the success rate was 100.00% in the observation group. There was no significant difference in the success rate between the two groups (*p* > 0.05), as shown in Figure 1.

### Comparison of Implant Deviation Between Two Groups

**Figure 2:**
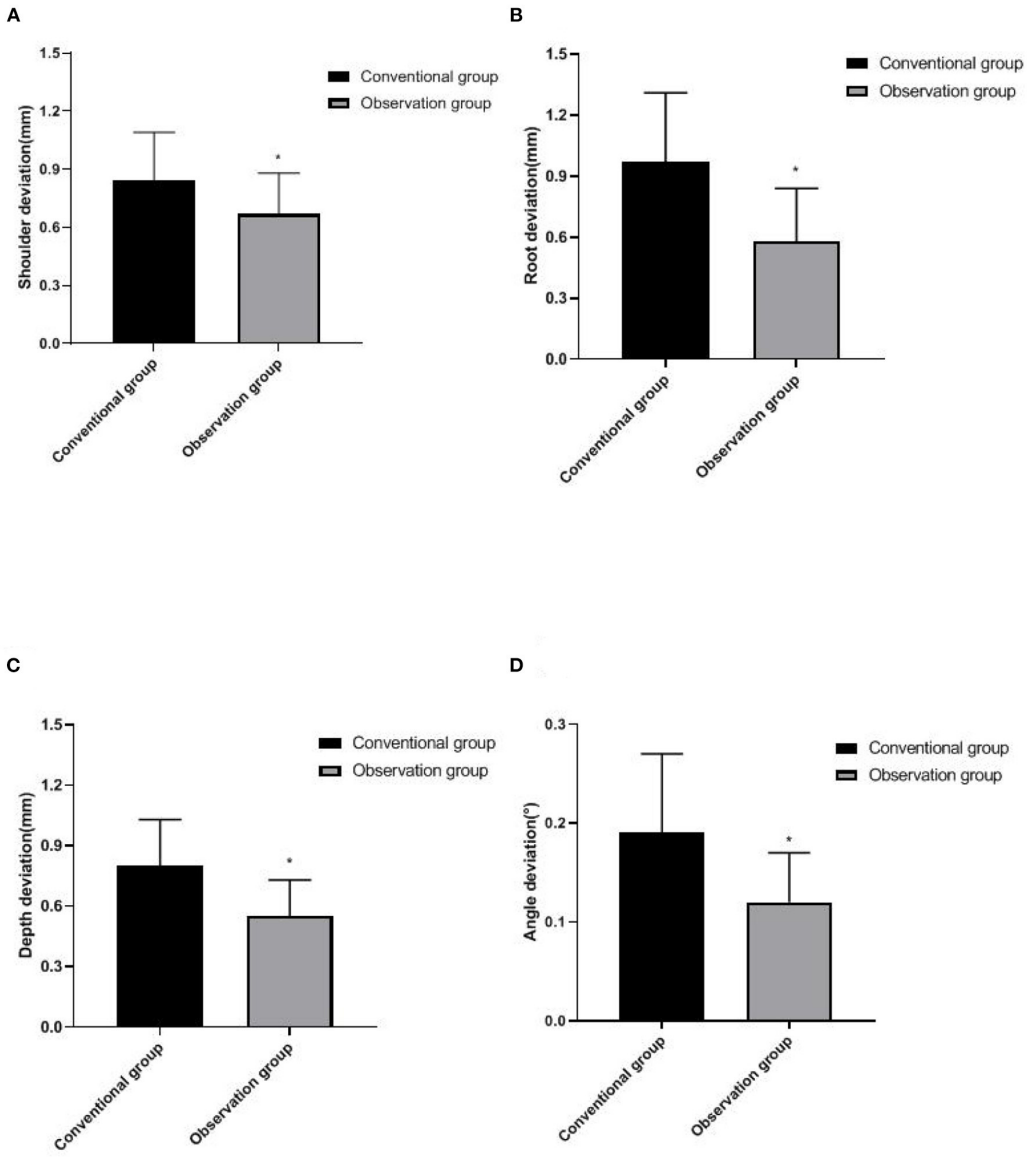
Comparison of implant deviation between two groups. **(A)** Shoulder deviation, **(B)** Root deviation, **(C)** Depth deviation, and **(D)** Angle deviation. Compared with the conventional group, *p < 0.05.

The implant deviation values in the observation group were all lower than those in the conventional group (*p* < 0.05), as shown in Figure 2.

### Comparison of Periodontal Index Between Two Groups

**Figure 3:**
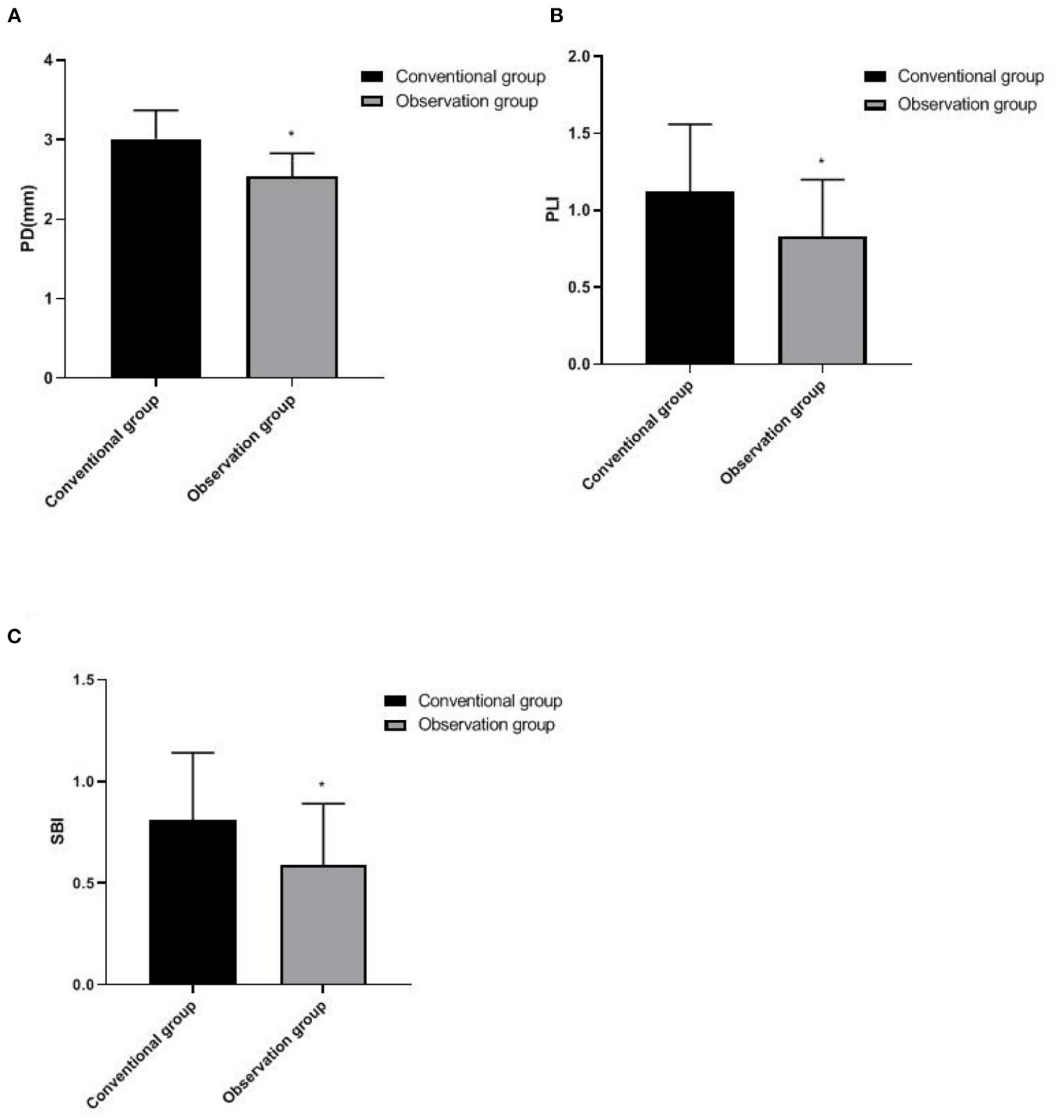
Comparison of periodontal index between two groups. **(A)** PD, **(B)** PLI, and **(C)** SBI. Compared with the conventional group, *p < 0.05.

PD, PLI, and SBI in the observation group were all lower than those in the conventional group (*p* < 0.05), as shown in Figure 3.

### Comparison of Absorption of Labial Bone Plate Between Two Groups

**Figure 4:**
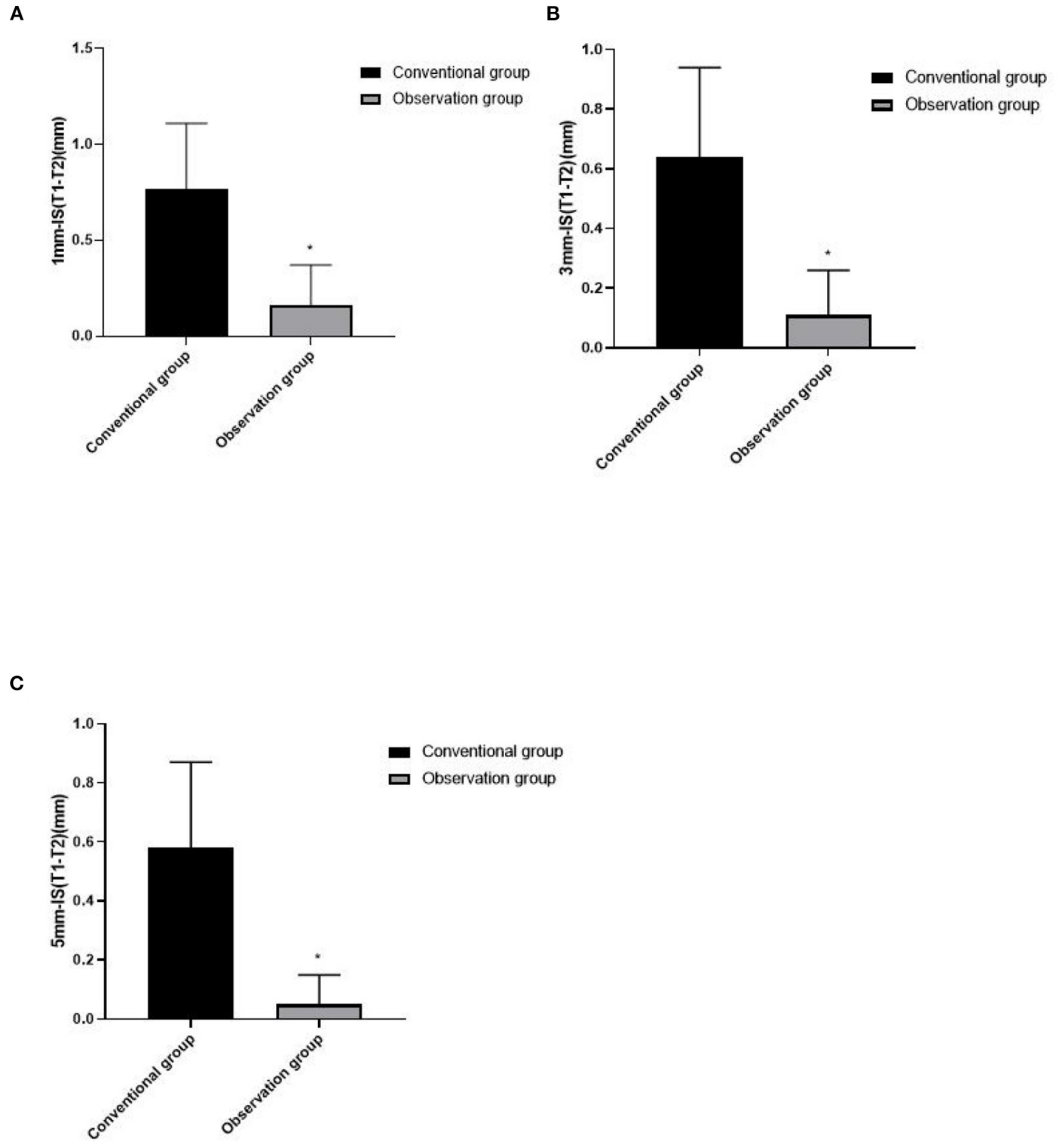
Comparison of absorption of labial bone plate between two groups. **(A)** 1mm-IS(T1-T2), **(B)** 3mm-IS(T1-T2), and **(C)** 5mm-IS(T1-T2). Compared with the conventional group, *p < 0.05.

The absorption value of labial bone plate in the observation group was all lower than those in the conventional group (*p* < 0.05), as shown in Figure 4.

### Comparison of Complications Between the Two Groups

The total incidence of complications in the observation group (5.88%) was lower than that in the conventional group (19.61%) (*p* < 0.05), as shown in Table 2.

**Table 2:** Comparison of complications between two groups (*n*, %).

**Group**	**Periimplant inflammation**	**Crown fracture**	**Gingival swelling**	**Neuropathic pain**	**Malocclusion**	**Total incidence rate**
Conventional group (*n* = 51)	2 (3.92%)	1 (1.96%)	5 (9.80%)	1 (1.96%)	1 (1.96%)	10 (19.61%)
Observation group (*n* = 51)	0 (0.00%)	0 (0.00%)	2 (3.92%)	1 (1.96%)	0 (0.00%)	3 (5.88%)
*χ^2^/t-*value						4.320
*p*-value						0.038

### Comparison of Aesthetic Effects Between the Two Groups

**Figure 5:**
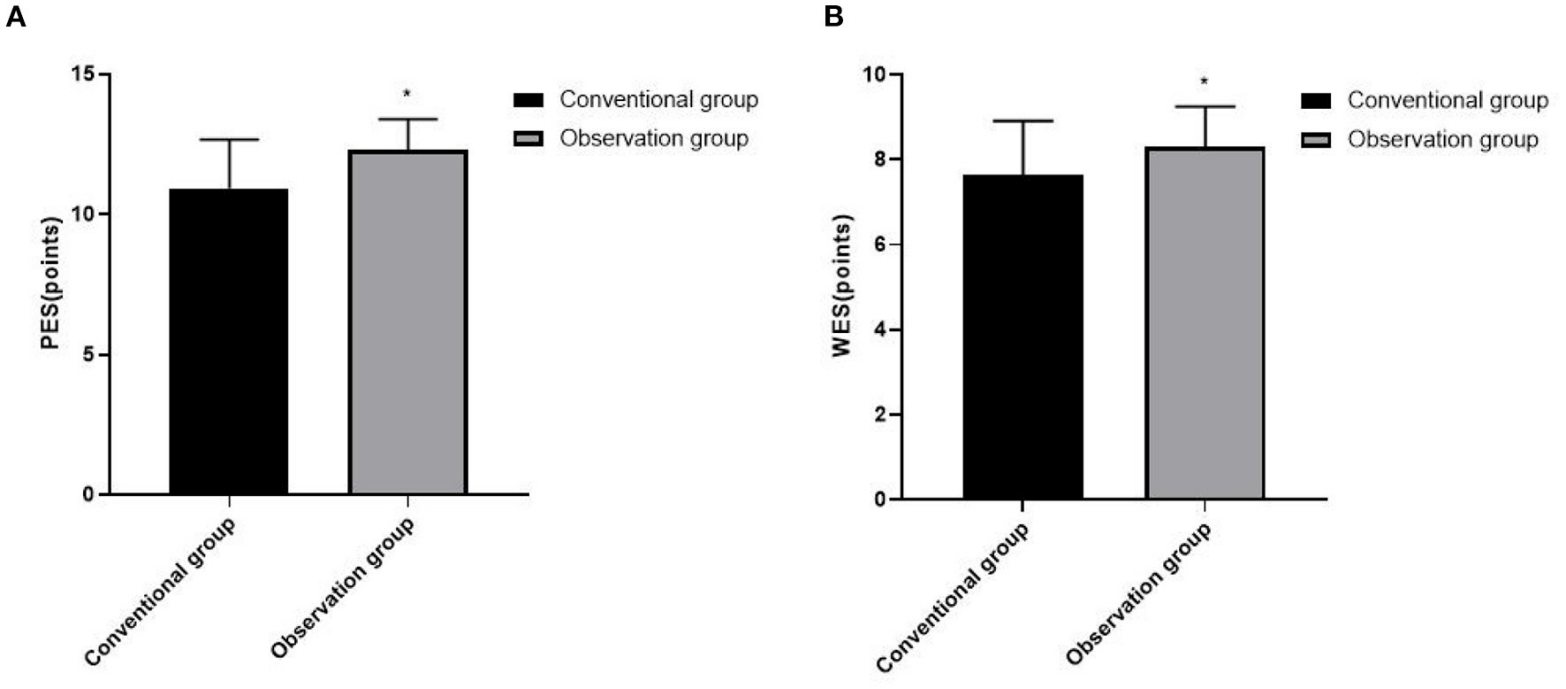
Comparison of aesthetic effects between the two groups. **(A)** PES and **(B)** WES. Compared with the conventional group, *p < 0.05.

The PES and WES in the observation group were higher than those in the conventional group (*p* < 0.05), as shown in Figure 5.

### Comparison of Satisfaction Between the Two Groups

The total satisfaction in the observation group (92.16%) was higher than that in the conventional group (76.47%) (*p* < 0.05), as shown in Table 3.

**Table 3:** Comparison of satisfaction between the two groups (*n*, %).

**Group**	**Very satisfied**	**Satisfied**	**Dissatisfied**	**Total satisfaction**
Conventional group (*n* = 51)	22 (43.14%)	17 (33.33%)	12 (23.53%)	39 (76.47%)
Observation group (*n* = 51)	28 (54.90%)	19 (37.25%)	4 (7.84%)	47 (92.16%)
*χ^2^/t-*value				4.744
*p-*value				0.029

### Typical Cases

**Figure 6:**
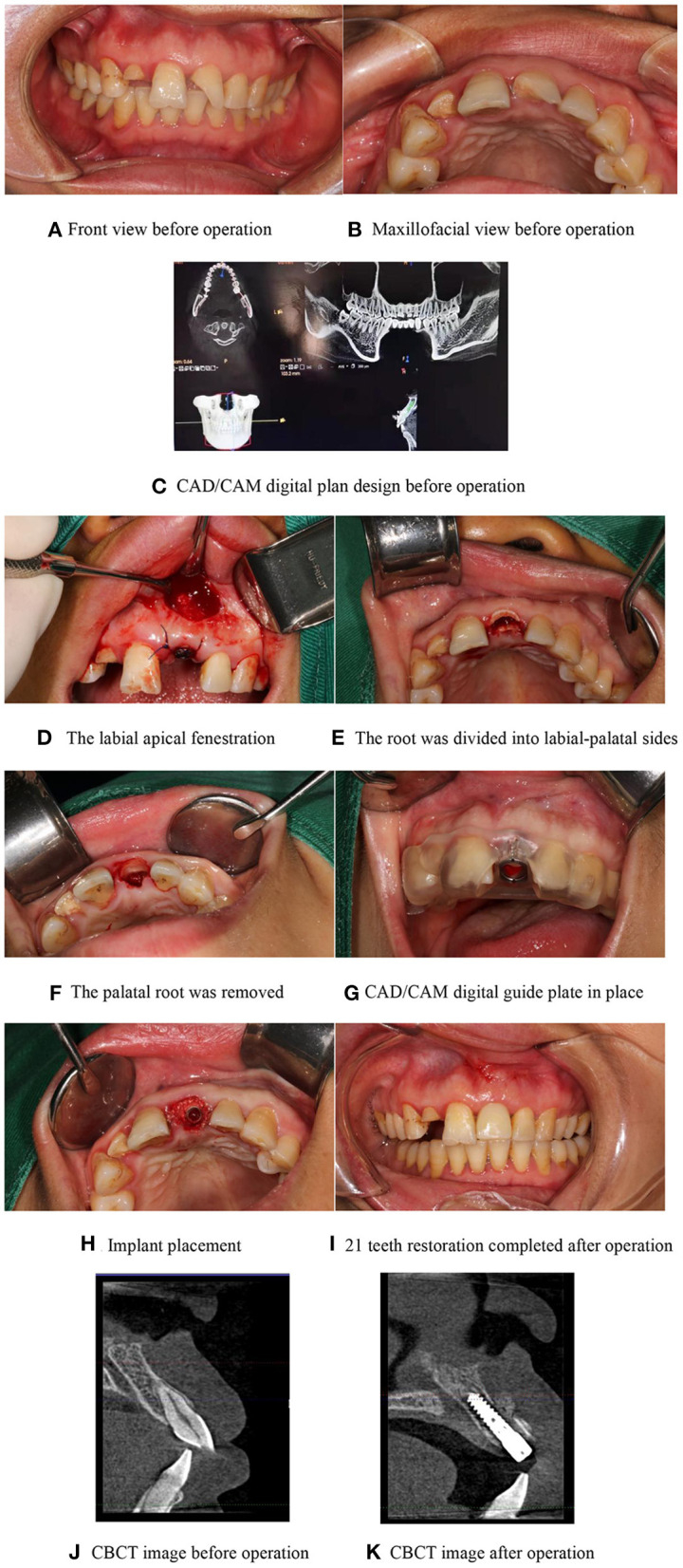
Typical cases. **(A)** Front view before operation, **(B)** Maxillofacial view before operation, **(C)** CAD/CAM digital plan design before operation, **(D)** The labial apical fenestration, **(E)** The root was divided into labial-palatal sides, **(F)** The palatal root was removed, **(G)** CAD/CAM digital guide plate in place, **(H)** Implant placement, **(I)** 21 teeth restoration completed after operation, **(J)** CBCT image before operation, and **(K)** CBCT image after operation.

A female patient who aged 35 years suffered from a traumatic injury to her upper left anterior tooth a month ago and came to our hospital for treatment because of the impact on the aesthetics of the teeth. The patient was physically healthy, denied a history of systemic disease, and denied a history of drug allergy. Extraoral examination showed that the patient's facial appearance was symmetrical, her mouth opening was normal, there was no buzzing, murmur, or tenderness in the temporomandibular joint area, and no enlarged lymph nodes were palpable in the submandibular, submental, and neck areas. Intraoral examination showed that the 21 mesial crown fracture defect, mesial and palatal side involving 1 mm subgingival, no loosening, tapping [-], gingival biologic pattern of medium thick gingival, root surface convexity consistent with the contralateral tooth of the same name, and median laughter line. Before the operation, CBCT showed that the 21 labial bone plate was continuous and complete, with a thickness of about 0.8 mm, the root of the tooth was close to the labial bone plate, and the height of apical bone was > 4 mm. It was diagnosed that 21 crown-roots were broken. The CAD/CAM guides combined with SST were performed on the patient. After the operation, the tooth was well repaired. The color of the restoration was similar to that of the adjacent teeth. The restoration was in harmony with the whole teeth, the gingival was in good condition, and the bone level at the edge of the implant was stable. The patient was satisfied with the restoration effect, as shown in Figures 6 (A–K).

## Discussion

In recent years, with the improvement in economic level, people pay more and more attention to the problem of tooth health, and people are eager to repair the defect of anterior teeth aesthetic area and achieve ideal aesthetic effect. Immediate implantation, as a common clinical dental restoration method, is maturing in the development of artificial implantation technology, but its surgical accuracy and density can no longer meet the needs of patients. Because of the thin labial bone wall and thick palatal bone wall in the aesthetic area of anterior teeth, it is easy to cause the drill needle to shift to labial side when preparing the implant socket. In addition, immediate implantation cannot avoid alveolar bone absorption, especially in patients with thin labial bone plate, the probability of soft tissue retraction is obviously increased, resulting in a series of complications (10). Therefore, determining the three-dimensional position of the implant and maintaining the thickness of the labial bone wall play a vital role in the restoration of the aesthetic area of the anterior teeth, which has become an urgent problem to be solved in the current implant field.

With the emergence of digital information technology, the application of CAD/CAM guide plate in clinic has been widely recognized. In this study, 102 patients with immediate implants were treated with CAD/CAM guide combined with SST, and finally, the success rate was 100.00%, and the deviation value of implants was small. The possible reason is that by importing the data derived from CBCT into professional software, deeply analyzing the three-dimensional data in the oral cavity and reconstructing the three-dimensional jaw model, the type, location, and depth of the implant can be determined (11). The dental implant guide plate manufactured by CAD/CAM greatly defines the angle and direction of implant implantation, which can provide more accurate positioning for immediate implant operation, significantly reduce the deviation value of empirical operation in space position and then improve the precision of implant and increase the probability of successful implant (12). In addition, during the implementation of SST, the surgeon divides the root of the tooth, extracts the root of the palatal tooth, and retains the dental slice shield, which is beneficial to enhance the resistance of the labial bone plate to external forces and maintain the blood supply of periodontal ligament to the labial bone plate (13). SST is a simple surgical procedure, which can preserve as many periodontal ligament cells as possible, reduce trauma of soft tissue and bone plate, reduce the risk of alveolar bone absorption and collapse, and play a positive role in increasing the stability of implants (14).

We found that the absorption value of labial bone plate and the total incidence of complications in the observation group were lower than those in the conventional group. The results suggest that the application of CAD/CAM guide plate combined with SST in immediate implantation of anterior teeth aesthetic area can reduce the degree of bone resorption and complications. After applying CAD/CAM guide plate and SST in immediate implantation, it can induce the secretion of growth factors related to periodontal tissue regeneration and promote bone formation and reconstruction. At the same time, it can avoid squeezing the labial bone plate, increase blood supply, change the way that alveolar ridge bears stress, and stimulate the proprioceptor of periodontal membrane of anterior teeth, thereby reducing the absorption of labial bone plate. In the process of traditional immediate implant operation, the angle change and shaking of twist drill may lead to complications such as crown fracture and gingival swelling after operation, which will further affect the restoration effect. Compared with the traditional immediate implantation, SST based on CAD/CAM guide plate can prevent the twist drill from producing excessive impact and vibration by guiding the operator to determine the three-dimensional position of the implant and reduce the operation requirement of angle change, so that the implant can be implanted into the missing tooth more accurately and in a reasonable position. This surgical scheme has the advantages of less trauma and low risk, which can prevent the implant from touching the important nerves and blood vessels of periodontal tissue and reduce the occurrence of complications such as bleeding and peri implant inflammation. We also found that the application of CAD/CAM guide plate combined with SST can reduce PD, PLI, and SBI and promote periodontal health. Sun's team reported that the PD, PLI, and SBI in SST group were significantly lower than those in traditional group (15). After the implementation of SST, we applied CAD/CAM guide plate to implant, which made the oral structure, operation process, and other information clearer and more transparent, greatly reduced the surgical trauma, avoided damaging the periodontal tissue, and was more conducive to improve the postoperative periodontal environment.

For patients with defects in the aesthetic area of anterior teeth, they often have higher aesthetic restoration requirements. Not only the white aesthetics of restoration are the key part of this area, but also the pink aesthetics such as the shape, texture, color, and outline of gums are the focus of oral aesthetics (16). In this study, CAD/CAM guide plate combined with SST can improve the aesthetics of immediate implantation in the aesthetic area of anterior teeth and obtain higher satisfaction. In the process of CAD/CAM guide plate design, the operator and the patient communicate accurately and intuitively. After doctors know the patient's repair needs, they can extract oral information through CBCT, reconstruct the three-dimensional jaw image, and measure the available bone width and the available bone height in the missing tooth area, so as to determine the three-dimensional position of the implant. This can obtain good initial stability, which is beneficial to achieve accurate and beautiful implant restoration effect (17). The design and manufacture of CAD/CAM guide plate plays a certain restrictive role in implant operation, which is beneficial to avoid the poor implant position caused by the operator's lack of experience, and makes the tooth position structure after operation close to the normal physiological state, thus meeting the aesthetic requirements of immediate implant patients (18). SST is beneficial to preserve the amount of labial alveolar bone, maintain the thickness and contour of bone plate, improve the shape and position of gingival margin, and provide conditions for the regeneration of soft and hard tissues, so that the immediate implant site can obtain a stable aesthetic effect (19, 20). SST is easy to operate, less surgical trauma, no excessive surgical intervention, and relatively low repair cost, which is easily accepted by patients (21).

It is worth noting that although the implementation of CAD/CAM guide plate has high accuracy, there is still some deviation in clinical application, which is the result of superposition of many factors, and the deviation may occur in any operation. At the same time, SST has the limitation of narrow indications, and it cannot be applied to cases with chronic apical infection. Additionally, the development of SST still has some problems such as tooth position, tooth outcome, curved root, and so on, and there may be potential risks in practical application. Therefore, it is necessary for clinicians to master professional knowledge and clinical skills, minimize the controllable deviation factors during implantation, and carefully implement SST to protect dental tablets.

## Conclusion

To sum up, the application of CAD/CAM guide plate combined with SST in immediate implantation of anterior teeth aesthetic area has a good effect, which can improve the accuracy of implantation, improve the periodontal environment, reduce bone resorption, reduce complications, improve aesthetics, and have high patient satisfaction. The results of this study still need to be further verified by a larger sample size data, and the long-term effects need to be discussed.

## Data Availability Statement

The original contributions presented in the study are included in the article/Supplementary Materials, further inquiries can be directed to the corresponding author.

## Author Contributions

ZW and JL mainly responsible for data statistics and paper writing. XW and NW are mainly responsible for the inclusion and treatment of cases and the detection of results. MT is mainly responsible for the guidance of the whole study. All authors contributed to the article and approved the submitted version.

## Conflict of Interest

The authors declare that the research was conducted in the absence of any commercial or financial relationships that could be construed as a potential conflict of interest.

## Publisher's Note

All claims expressed in this article are solely those of the authors and do not necessarily represent those of their affiliated organizations, or those of the publisher, the editors and the reviewers. Any product that may be evaluated in this article, or claim that may be made by its manufacturer, is not guaranteed or endorsed by the publisher.
