# Supplementary figures and images for: Effect of CAD/CAM Guide Plate Combined With Socket-Shield Technique in Immediate Implantation of Anterior Teeth Aesthetic Area and Its Influence on Aesthetics
Source: Front Surg. 2022 Jan 25;8:833288. doi: 10.3389/fsurg.2021.833288 (PMC8821654; doi:10.3389/fsurg.2021.833288)

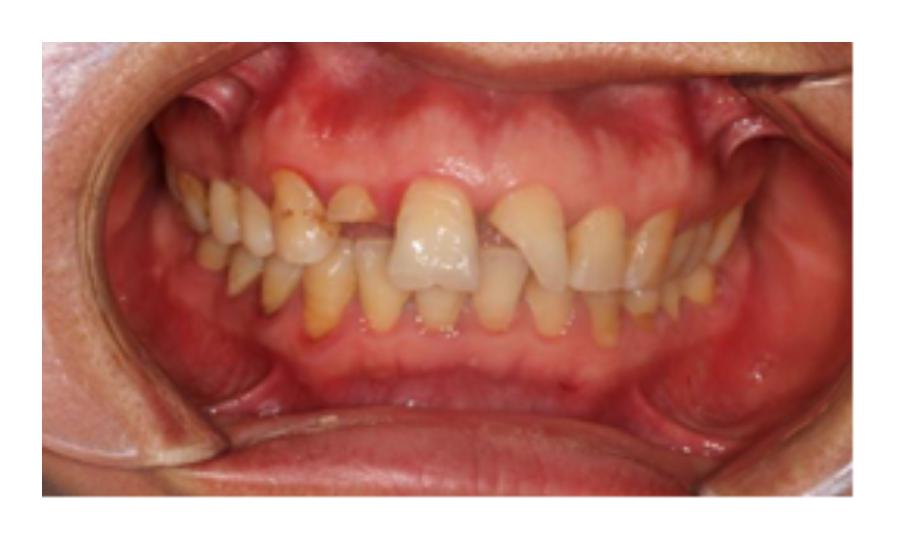

Supplement: Supplementary file 1 [file Data_Sheet_1.ZIP › Original Figure/Figure 6a.jpg]

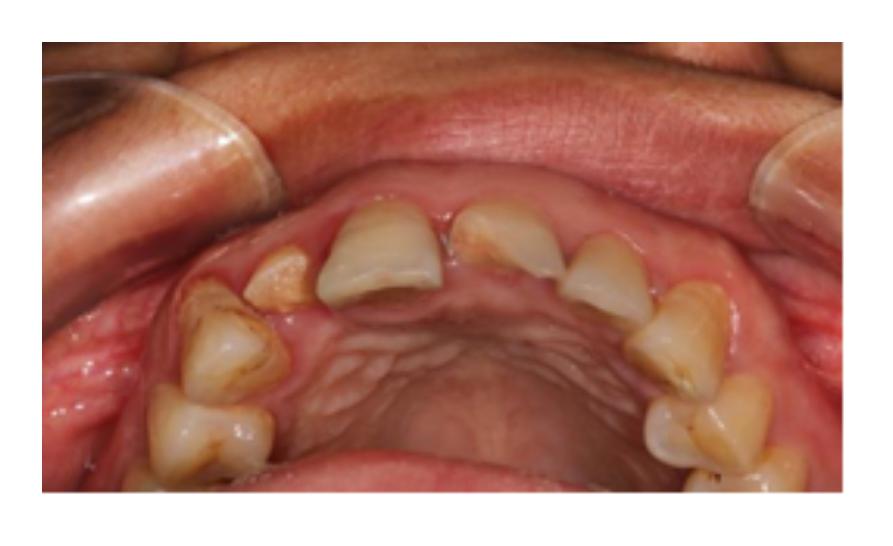

Supplement: Supplementary file 1 [file Data_Sheet_1.ZIP › Original Figure/Figure6b.jpg]

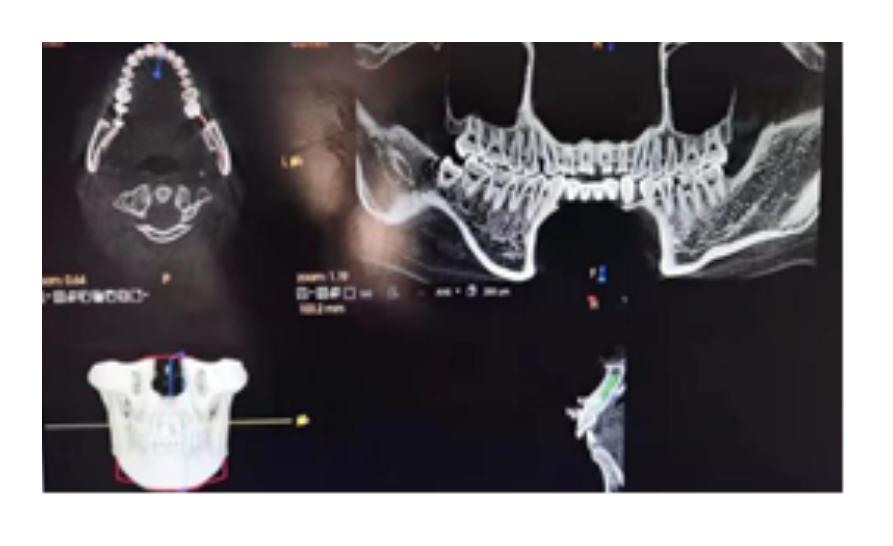

Supplement: Supplementary file 1 [file Data_Sheet_1.ZIP › Original Figure/Figure6c.jpg]

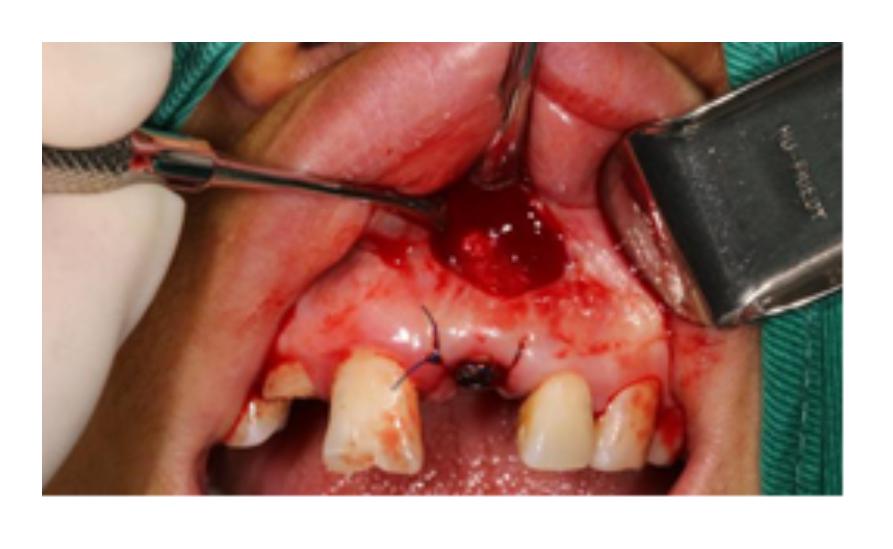

Supplement: Supplementary file 1 [file Data_Sheet_1.ZIP › Original Figure/Figure6d.jpg]

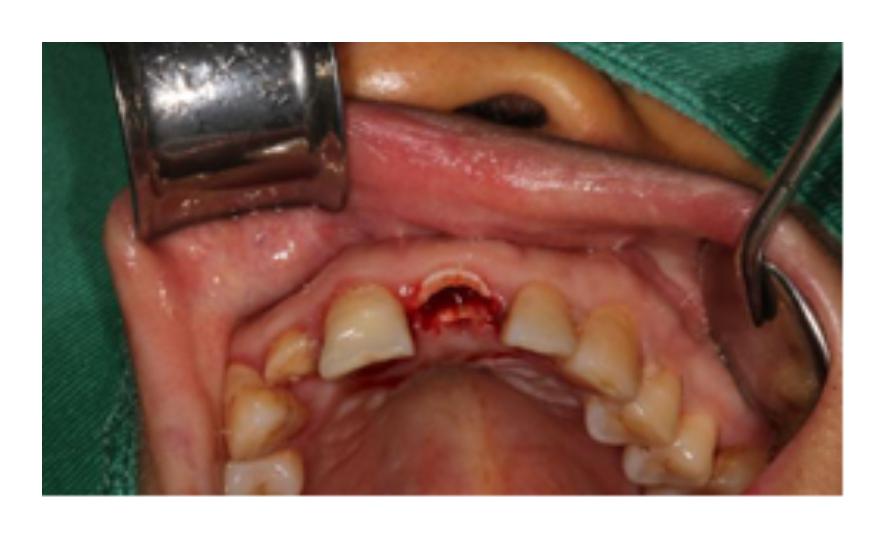

Supplement: Supplementary file 1 [file Data_Sheet_1.ZIP › Original Figure/Figure6e.jpg]

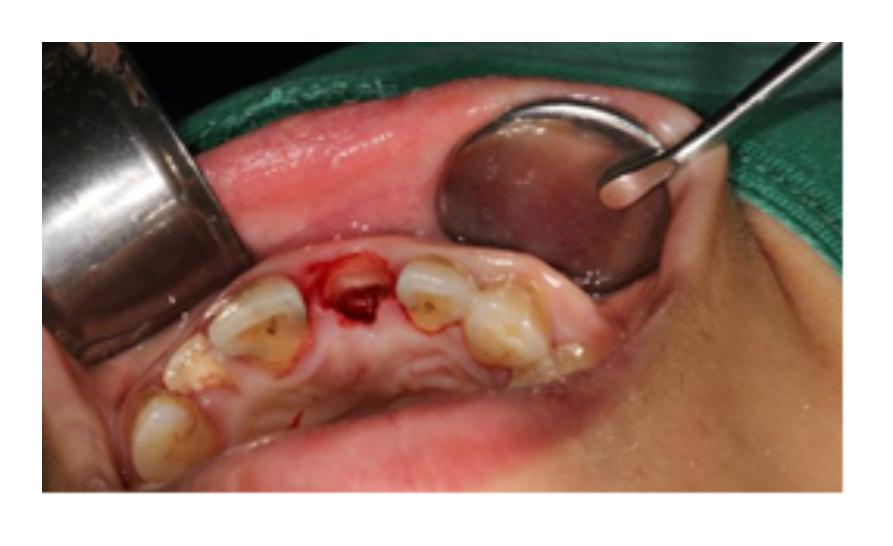

Supplement: Supplementary file 1 [file Data_Sheet_1.ZIP › Original Figure/Figure6f.jpg]

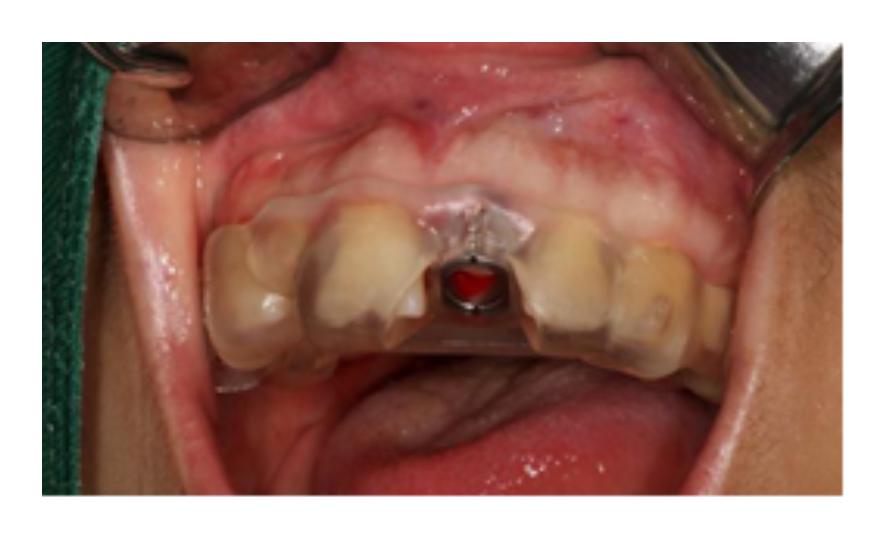

Supplement: Supplementary file 1 [file Data_Sheet_1.ZIP › Original Figure/Figure6g.jpg]

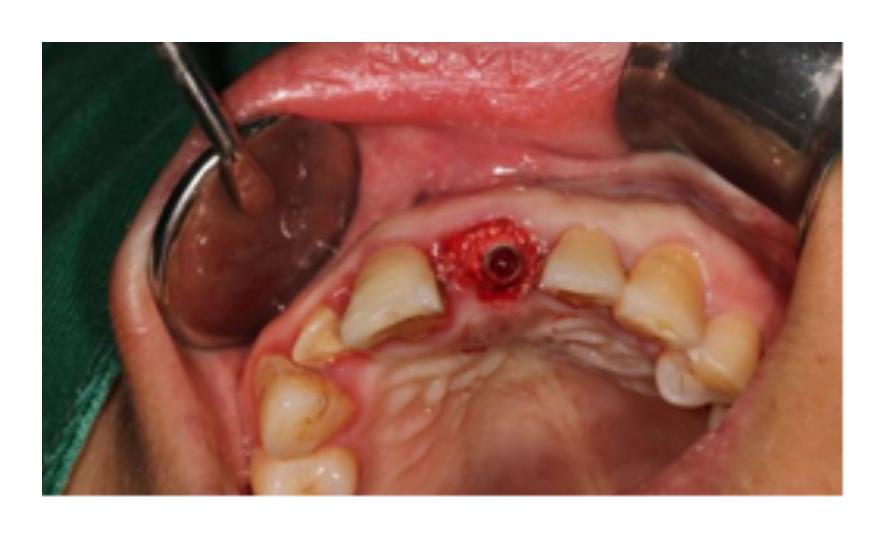

Supplement: Supplementary file 1 [file Data_Sheet_1.ZIP › Original Figure/Figure6h.jpg]

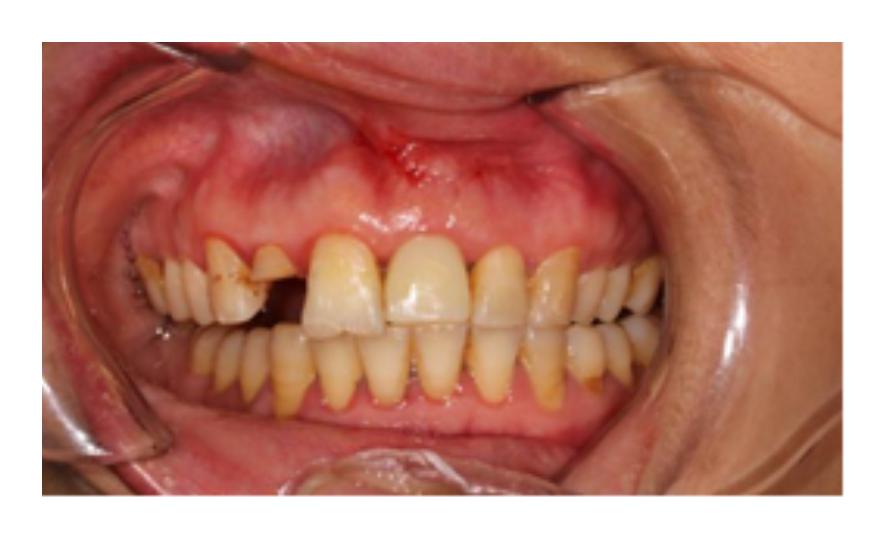

Supplement: Supplementary file 1 [file Data_Sheet_1.ZIP › Original Figure/Figure6i.jpg]

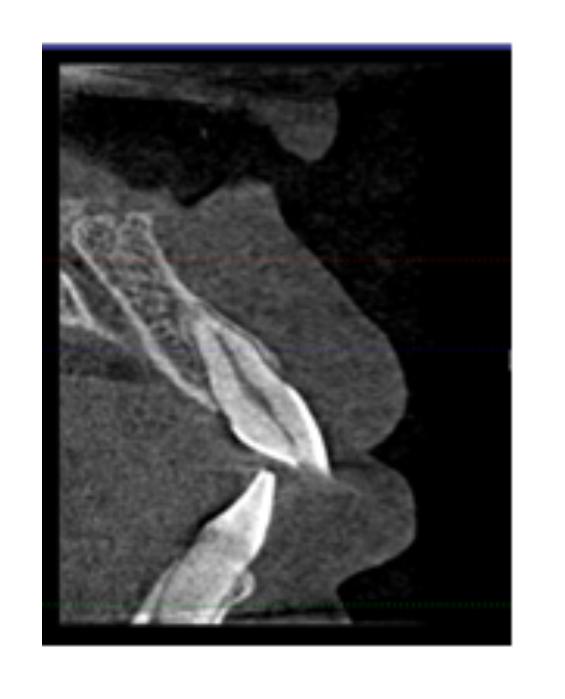

Supplement: Supplementary file 1 [file Data_Sheet_1.ZIP › Original Figure/Figure6j.jpg]

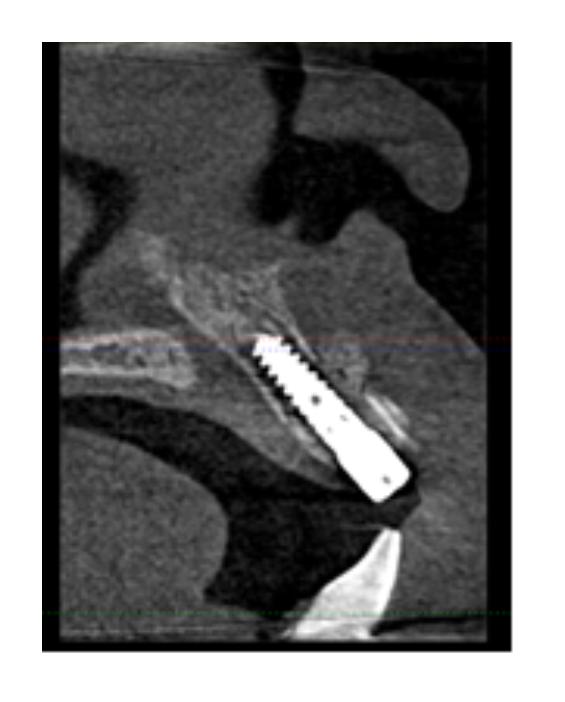

Supplement: Supplementary file 1 [file Data_Sheet_1.ZIP › Original Figure/Figure6k.jpg]
